# Correction Method for the Observed Global Navigation Satellite System Ultra-Rapid Orbit Based on Dilution of Precision Values

**DOI:** 10.3390/s18113900

**Published:** 2018-11-12

**Authors:** Qianxin Wang, Chao Hu, Ya Mao

**Affiliations:** 1 NASG Key Laboratory of Land Environment and Disaster Monitoring, China University of Mining and Technology, Xuzhou 221116, China; maoya1994@cumt.edu.cn; 2 School of Environment Science and Spatial Informatics, China University of Mining and Technology, Xuzhou 221116, China; 3 Satellite Positioning for Atmosphere, Climate and Environment Research Centre, School of Science, Mathematical and Geospatial Sciences, RMIT University, Melbourne 3001, Australia

**Keywords:** observed ultra-rapid orbit, orbit determination, dilution of precision, amplification factor, orbit correction

## Abstract

For ultra-rapid orbits provided by the Global Navigation Satellite System (GNSS), the key parameters, accuracy and timeliness, must be taken into consideration in real-time and near real-time applications. However, insufficient observations in later epochs appear to generate low accuracy in observed orbits, for which a correlation between the Dilution of Precision (DOP) of the orbit parameters and their accuracy is found. To correct the observed GNSS ultra-rapid orbit, a correction method based on the DOP values is proposed by building the function models between DOP values and the orbit accuracy. With 10-day orbit determination experiments, the results show that the observed ultra-rapid-orbit errors, generated by insufficient observations, can be corrected by 12–22% for the last three hours of the observed orbits. Moreover, considering the timeliness constraints in ultra-rapid-orbit determination, a DOP amplification factor is defined to weight the contribution of each tracking station and optimize the station distribution in the orbit determination procedure. Finally, six schemes are designed to verify the method and strategy in determining the ultra-rapid orbit based on one-month observations. The orbit accuracy is found to decrease by 1.27–6.34 cm with increasing amplification factor from 5–20%. Thus, the observed ultra-orbit correction method proposed is ideal when considering accuracy and timeliness in ultra-rapid orbit determination.

## 1. Introduction

The analysis center (AC) of a Global Navigation Satellite System (GNSS) provides ultra-rapid, rapid, and final products and services, such as orbits and clocks, to GNSS users, of which the ultra-rapid orbit plays an important role in real-time and near real-time applications. Because the accuracy of the ultra-rapid orbit affects directly the ambiguity resolution [1] and the results of precise point positioning [2], there is a strict accuracy requirement on ultra-rapid orbits from ACs. For instance, the International GNSS Monitoring and Assessment Service (iGMAS) lists in detail the observed and predicted accuracies of multi-GNSS ultra-rapid orbits [3]. However, the three-dimensional root-mean-square errors (3D RMS) of GPS ultra-rapid predicted orbits within the 6- and 24-h periods may be up to 41.7 mm and 80.2 mm, respectively (IGS mail 6053), which lags behind the final precise orbit of the International GNSS Service (IGS) and does not meet with the high-precision requirements of GNSS users. Because the accuracy of ultra-rapid orbits is low, orbit prediction strategies [4], optimal prediction arcs [5], prediction time intervals [6], and the impact of Earth rotation parameters [7] were investigated by scholars to refine the orbit models and strategies. However, the research on ultra-rapid orbits is concerned mainly with the predicted values, whereas for the observed values, little has been done in detail. In addition, high-precision GNSS ultra-rapid orbits for different navigation satellite systems are prerequisite in the expansion to fast high-precision GNSS services with the flourishing development of the GNSS, which includes the “three-step” strategy of the BeiDou satellite system (BDS) [8].

Furthermore, timeliness also poses difficulties to GNSS ultra-rapid products and GNSS applications. However, multiple GNSS developments [9] and tracking network extensions of IGS, iGMAS, and Multi-GNSS EXperiment (MGEX) present a great challenge to ACs in regard to timeliness [10,11].

To improve the computational efficiency of multi-GNSS solutions, different strategies have been proposed concerning parameter optimization, such as parameter elimination [12], carrier-range [13], algorithm Ambizap [14], and increasing solution intervals [15]. Moreover, the optimal tracking stations distributions were investigated based on the relationship between the orbit parameters and geometrical configuration [11,16,17]. However, timeliness and accuracy of GNSS ultra-rapid-orbit determination cannot be thoroughly solved in light of the obvious drawbacks in the methods and strategies mentioned above such as the neglect of parameter correlations [18], inconsistent intervals and references of products, and inaccurate function models. Therefore, given the uneven tracking stations distribution, the redundancy of observations, and the increasing number of GNSS satellites, timeliness and accuracy of the ultra-rapid orbits determination needs to be taken into consideration.

To solve these problems, it should be noted that timeliness and accuracy of ultra-rapid orbits are related to tracking observations directly. Furthermore, there are two main indices used in analyzing the contribution of tracking observations to parameter solutions; they are called the observation accuracy and the DOP [19], the latter representing the impact of the geometrical configuration between satellites and tracking stations on the orbit determination parameters [20,21]. Moreover, the optimal configuration of point positioning, based on the DOP values has been widely studied [22]. It assumes minimum DOP values of Earth’s core as values on Earth’s surface but is inconsistent with the orbit determination models. In addition, for some special situations, the optimal distributions of stations based on the DOP values were proposed to determine Earth rotation parameters [23] and the BeiDou geosynchronous orbit [16]. However, according to the related orbit determination experiments, it is suggested that the number of stations and their distribution are correlated with the accuracy of the orbit and its related parameters [24,25]. Therefore, the accuracy of orbital space state parameters (positions and velocities) of each epoch influenced by observations may be represented by a function based on the orbit determination geometrical configuration. To correct the observed ultra-rapid orbit obtained from the available observations, a function relating the orbit accuracy and the DOP values is constructed. For areas with redundant observations, a strategy based on the minimum DOP values to optimize the observations may take timeliness and accuracy of ultra-rapid orbits into consideration more effectively. An effective way to improve accuracy and timeliness of the ultra-rapid orbit is achieved by making full use of the DOP values of the orbital state parameters in the ultra-rapid-orbit determination.

In this study, based on the low-accuracy observed ultra-rapid orbit generated by the available observations in the latter arcs and its impacts on the predicted parts, a function model is proposed that uses the correlation between orbit parameter accuracies and DOP values. With a highly accurate prediction of the DOP values, the observed orbit accuracy is improved with this orbit correction method. In addition, considering timeliness and accuracy of the ultra-rapid orbit, an optimized orbit determination strategy is discussed in terms of DOP values. Section 2 discusses the principle of the orbit correction method. Section 3 provides an analysis of the accuracy of current observed ultra-rapid orbit and its predicted parts. The experiments on orbit corrections and the contribution of tracking stations and the optimization of their distribution based on DOP values of ultra-rapid orbits are also presented in Section 3. Section 4 gives the experimental analyses of ultra-rapid orbit determination. Finally, conclusions and prospects are presented in Section 5.

## 2. Principle of Orbit Correction Method

Given the analyses in Section 3.1, the DOP values of orbit parameters were found to have a high correlation with orbit accuracy, especially when observations are sufficient. Therefore, in the last arcs of the observed ultra-rapid orbit, the accuracy of the orbit influenced by the observations may be corrected indirectly by the corresponding DOP values as detailed below.

Assume the observation equation of one epoch is [11]:

(1) $$V(t_{i})=L(t_{i})-A(t_{i})X(t_{i}),\;$$

where *i* represents the *t_i_*-th epoch, ***V***(*t_i_*) the residuals of the equation, ***X***(*t_i_*) the orbit parameters (positions and velocities), ***L***(*t_i_*) the observations, and ***A***(*t_i_*) the design matrix expressed as [10]:

(2) $$A(t_{i})=diag\left(\begin{array}{ccccc} a_{1} & a_{2} & \cdots & a_{m-1} & a_{m} \end{array}\right),\;$$

(3) $$a_{m}=\left[\begin{array}{cccccc} \frac{\partial \rho_{m,1}^{s_{m}-r_{1}}}{\partial x_{m}^{s_{m}}} & \frac{\partial \rho_{m,1}^{s_{m}-r_{1}}}{\partial y_{m}^{s_{m}}} & \frac{\partial \rho_{m,1}^{s_{m}-r_{1}}}{\partial z_{m}^{s_{m}}} & \frac{\partial \rho_{m,1}^{s_{m}-r_{1}}}{\partial {\dot{x}}_{m}^{s_{m}}} & \frac{\partial \rho_{m,1}^{s_{m}-r_{1}}}{\partial {\dot{y}}_{m}^{s_{m}}} & \frac{\partial \rho_{m,1}^{s_{m}-r_{1}}}{\partial {\dot{z}}_{m}^{s_{m}}}\\ \frac{\partial \rho_{m,2}^{s_{m}-r_{2}}}{\partial x_{m}^{s_{m}}} & \frac{\partial \rho_{m,2}^{s_{m}-r_{2}}}{\partial y_{m}^{s_{m}}} & \frac{\partial \rho_{m,2}^{s_{m}-r_{2}}}{\partial z_{m}^{s_{m}}} & \frac{\partial \rho_{m,2}^{s_{m}-r_{2}}}{\partial {\dot{x}}_{m}^{s_{m}}} & \frac{\partial \rho_{m,2}^{s_{m}-r_{2}}}{\partial {\dot{y}}_{m}^{s_{m}}} & \frac{\partial \rho_{m,2}^{s_{m}-r_{2}}}{\partial {\dot{z}}_{m}^{s_{m}}}\\ \vdots & \vdots & \vdots & \vdots & \vdots & \vdots \\ \frac{\partial \rho_{m,n}^{s_{m}-r_{n}}}{\partial x_{m}^{s_{m}}} & \frac{\partial \rho_{m,n}^{s_{m}-r_{n}}}{\partial y_{m}^{s_{m}}} & \frac{\partial \rho_{m,n}^{s_{m}-r_{n}}}{\partial z_{m}^{s_{m}}} & \frac{\partial \rho_{m,n}^{s_{m}-r_{n}}}{\partial {\dot{x}}_{m}^{s_{m}}} & \frac{\partial \rho_{m,n}^{s_{m}-r_{n}}}{\partial {\dot{y}}_{m}^{s_{m}}} & \frac{\partial \rho_{m,n}^{s_{m}-r_{n}}}{\partial {\dot{z}}_{m}^{s_{m}}} \end{array}\right]$$

where *m* and *n* denote the number of satellites and stations, respectively, $\frac{\partial \rho_{m,n}^{s_{m}-r_{n}}}{\partial x_{m}^{s_{m}}},\frac{\partial \rho_{m,n}^{s_{m}-r_{n}}}{\partial y_{m}^{s_{m}}},\frac{\partial \rho_{m,n}^{s_{m}-r_{n}}}{\partial z_{m}^{s_{m}}}$ and $\frac{\partial \rho_{m,n}^{s_{m}-r_{n}}}{\partial {\dot{x}}_{m}^{s_{m}}},\frac{\partial \rho_{m,n}^{s_{m}-r_{n}}}{\partial {\dot{y}}_{m}^{s_{m}}},\frac{\partial \rho_{m,n}^{s_{m}-r_{n}}}{\partial {\dot{z}}_{m}^{s_{m}}}$ denote the partial derivatives of the geometric distance between satellite *s_m_* and station *r_n_* with reference to the satellite positions and velocities as in [26]:

(4) $$\begin{array}{l} \left(\frac{\partial \rho_{m,n}^{s_{m}-r_{n}}}{\partial x_{m}^{s_{m}}},\frac{\partial \rho_{m,n}^{s_{m}-r_{n}}}{\partial y_{m}^{s_{m}}},\frac{\partial \rho_{m,n}^{s_{m}-r_{n}}}{\partial z_{m}^{s_{m}}},\frac{\partial \rho_{m,n}^{s_{m}-r_{n}}}{\partial {\dot{x}}_{m}^{s_{m}}},\frac{\partial \rho_{m,n}^{s_{m}-r_{n}}}{\partial {\dot{y}}_{m}^{s_{m}}},\frac{\partial \rho_{m,n}^{s_{m}-r_{n}}}{\partial {\dot{z}}_{m}^{s_{m}}}\right)\\ =\left(\frac{x_{m}^{s_{m}}-x_{n}^{r_{n}}}{\rho_{m,n}^{0}},\frac{y_{m}^{s_{m}}-y_{n}^{r_{n}}}{\rho_{m,n}^{0}},\frac{z_{m}^{s_{m}}-z_{n}^{r_{n}}}{\rho_{m,n}^{0}},\frac{\Delta t(x_{m}^{s_{m}}-x_{n}^{r_{n}})}{\rho_{m,n}^{0}(t_{r})},\frac{\Delta t(y_{m}^{s_{m}}-y_{n}^{r_{n}})}{\rho_{m,n}^{0}(t_{r})},\frac{\Delta t(y_{m}^{s_{m}}-y_{n}^{r_{n}})}{\rho_{m,n}^{0}(t_{r})}\right) \end{array}$$

where *t_r_* is the signal reception time and ∆*t* the time taken for the signal to go from satellite to station.

From Equation (1), the cofactor matrix can be expressed as:

(5) $$Q(t_{i})={\left(A^{T}(t_{i})PA(t_{i})\right)}^{-1},\;$$

where ***P*** is the weight matrix of the observations, which is elevation-dependent for observations below 30° as in [26]; the observations below 10° are deleted when using Equation (1). Then, the DOP value of the *k*-th parameter is:

(6) $$DOP{\left(t_{i}\right)}_{k}=\sqrt{Q{\left(t_{i}\right)}_{kk}}.\;$$

Hence, the initial precision of one parameter can be expressed as:

(7) $$\sigma_{k}=\sigma_{0}\cdot DOP{\left(t_{i}\right)}_{k},\;$$

where *σ*_0_ is related to the accuracy of observations.

To build the orbit correction function, the total DOP values up until the current epoch for one orbit parameter is:

(8) $$[DOP{\left(t_{i}\right)}_{k}]=\sqrt{{\left[DOP{\left(t_{i-1}\right)}_{k}\right]}^{2}+{\left(DOP{\left(t_{i}\right)}_{k}\right)}^{2}}.\;$$

Here $\left[DOP{\left(t_{i-1}\right)}_{k}\right]$ represents the total values of the *k*-th parameter between the first and *t_i_*_−1_ epoch.

Suppose that the parameter corrections of the orbital states of a satellite are *d**X***(*t_i_*), which can be expressed as functions of [***DOP***(*t_i_*)]; hence:

(9) $$dX(t_{i})=\left[\begin{array}{c} dX_{1}\\ dX_{2}\\ \vdots \\ dX_{6} \end{array}\right]=\left[\begin{array}{c} f([DOP{\left(t_{i}\right)}_{1}])\\ f([DOP{\left(t_{i}\right)}_{2}])\\ \vdots \\ f([DOP{\left(t_{i}\right)}_{6}]) \end{array}\right],\;$$

where *f*(·) is the orbit correction function, which is determined by the orbit length and interval. Moreover, *d**X***(*t_i_*) denotes the corrections for positions and velocities of a satellite as given in Equation (3). From the equations of the orbit determination, the DOP values of the orbit parameters of each epoch can be accurately acquired. Therefore, the function models between the orbit parameter corrections and the DOP values can be built based on different mathematical models, which should be selected before orbit correction. In this section, to clearly describe the correction method, the polynomial model chosen as an example to discuss is:

（10） $$\left[\begin{array}{c} f([DOP{\left(t_{i}\right)}_{1})]\\ f([DOP{\left(t_{i}\right)}_{2})]\\ \vdots \\ f([DOP{\left(t_{i}\right)}_{6})] \end{array}\right]=\left[\begin{array}{ccccc} \left[DOP{\left(t_{i}\right)}_{k}\right] & {\left[DOP{\left(t_{i}\right)}_{k}\right]}^{2} & \cdots & {\left[DOP{\left(t_{i}\right)}_{k}\right]}^{d} & 1\\ \left[DOP{\left(t_{i}\right)}_{k}\right] & {\left[DOP{\left(t_{i}\right)}_{k}\right]}^{2} & \cdots & {\left[DOP{\left(t_{i}\right)}_{k}\right]}^{d} & 1\\ \vdots & \vdots & \ddots & \vdots & \vdots \\ \left[DOP{\left(t_{i}\right)}_{k}\right] & {\left[DOP{\left(t_{i}\right)}_{k}\right]}^{2} & \cdots & {\left[DOP{\left(t_{i}\right)}_{k}\right]}^{d} & 1 \end{array}\right]\left[\begin{array}{c} \alpha_{1}\\ \alpha_{2}\\ \vdots \\ \alpha_{d}\\ \alpha_{0} \end{array}\right]+\left[\begin{array}{c} \xi_{1}\\ \xi_{2}\\ \vdots \\ \xi_{6} \end{array}\right]$$

where *α* represents the polynomial coefficient, *ξ* the fitting residual, and *d* the order of the polynomial function. In Equation (10), *d* should be selected based on the Akaike information criterion [27] to avoid polynomials of too-high order in different orbit determination solutions. Moreover, the maximum of *d* should be less than 9. The function between the orbit parameters corrections and the corresponding DOP values may be established by estimating the polynomial coefficients accurately. For a low accuracy of the last arc in the observed ultra-rapid orbit, the corrections may be acquired indirectly using Equation (9), which takes the DOP values as independent variables. Following [28] and the characteristics of the DOP values, the orders of the polynomial function should be calculated using the Akaike information criterion [27] to describe the trend well. We assume the DOP values for a satellite have the form:

(11) $$[\bar{DOP}{\left(t_{j}\right)}_{k}]=\theta_{0}+\theta_{1}t_{j}+\cdots +\theta_{b}t_{j}^{b}+e(t_{j}),$$

where *θ* denotes polynomial coefficient; *e* the model error, and *t_j_* the epoch number. Stacking all available DOP values as a vector, Equation (11) becomes:

(12) $$[\bar{DOP_{k}}]=G\theta +d[\bar{DOP_{k}}],\;$$

where ***G*** represents the coefficient matrix, $d[{\bar{DOP}}_{k}]$ the residuals of the fitting models, and $\theta =[\begin{array}{ccccc} \theta_{0} & \theta_{1} & \theta_{2} & \cdots & \theta_{b} \end{array}]$^T^. Thus, the coefficient of the DOP function is:

(13) $$\hat{\theta }={\left(G^{T}G\right)}^{-1}G^{T}[{\bar{DOP}}_{k}],\;$$

Setting the current epoch as $t_{j}=0$, then, the predicted $\left[DOP{\left(t_{j}\right)}_{k}\right]$ is:

(14) $$[DOP{\left(t_{j}\right)}_{k}]={\hat{\theta }}_{0}.\;$$

Substituting the predicted DOP values into Equation (9) yields the orbit corrections and improves the orbit accuracy. However, the correction method mentioned concerning the observed ultra-rapid orbit is based on the optimal geometrical configuration between satellites and stations. Theoretically, the more tracking stations and the more uniform the distribution of the stations, the better the geometrical configuration is for orbit determination. However, because of timeliness of the ultra-rapid orbit, it is impossible to deal with all observations downloaded by the ACs. Hence, strategies for orbit determination, taking accuracy and timeliness into account, are discussed in the following sections.

## 3. Experiments Results of Ultra-Rapid Orbit Determination Correction

### 3.1. Accuracy Analysis of the Observed Ultra-Rapid Orbit

To serve real-time and near real-time users, the IGS began producing ultra-rapid-orbit products officially on November 2000, originally with updates every 12 h [29]. The update cycle was reduced to every 6 h starting April 2004, the update comprising 24-h observed orbit and 24-h predicted orbit services. At present, GNSS users can acquire combined GPS and GLONASS ultra-rapid orbit with a 3-h latency, whereas iGMAS provides a four-system ultra-rapid orbit (including Galileo and BeiDou). To analyze the accuracy of the ultra-rapid orbit fully, the products from Wuhan University (WHU, iGMAS AC) [30] and the German Research Center for Geosciences (GFZ, IGS AC) [31] are taken as references to calculate the orbit residuals. One-month of ultra-rapid orbits from day of year (DOY) 168 to 197, 2017 of WHU were selected in the orbit accuracy analysis. The residuals between the observed parts of the ultra-rapid and the rapid orbits were extracted. The corresponding average 3D RMSs, listed in Table 1, show that the accuracy of the observed ultra-rapid orbits decreases for all systems in the latter arcs of the observed parts, especially for the final 3 h. Moreover, note that the results of the 3D RMSs are calculated based on data from all satellites of each system for which the low accuracy of the observed parts is not suitable for all satellites involved based on the experiments.

To describe the orbit errors in more detail, the ultra-rapid orbit from WHU (whu19540_00.sp3, DOY 168, 2017) is set as an example. Corresponding to the ultra-rapid orbit of WHU, the GFZ rapid orbit (gbm19536.sp3) is selected as a reference to compare the orbit accuracy between different ACs. Figure 1 plots the 3D RMSs of orbits from the four satellite systems (GPS/GLONASS/Galileo/BeiDou) between WHU and GFZ during the last 3-h period, in which only the satellites with significant reduction in accuracy are extracted to highlight the trend.

In addition, to describe the impact of low accuracy of the observed part on the predicted orbit, the last 1-h of observed orbits of WHU are extracted to predict the 24-h multi-GNSS orbits. Similarly, the WHU rapid orbits are set as references to analyze the accuracy of predicted orbits, the 3D RMSs of which are listed in Table 2 at 2-h intervals during the first 6 h. The accuracy of the predicted orbits is also verified by one-month experiments. To show more detail concerning the accuracy of the predicted orbits based on the observed parts, WHU (whu19540_00.sp3) is extracted to fit the initial orbit and predict the 24-h multi-GNSS orbit. Next, the GFZ rapid orbit (gbm19540.sp3) is set as a reference to analyze the accuracy of the predicted orbit (see Figure 2).

From the accuracy of the observed and predicted parts of the ultra-rapid orbit, we conclude: (1) the accuracy of the observed ultra-rapid orbit is obviously reduced during the last 2–3-h period of the observed parts, especially for the BeiDou orbits; (2) the predicted part of the ultra-rapid orbit is significantly limited in real-time and near real-time applications as its accuracy is influenced by that of the observed part; (3) not all satellites have a similar reduction in accuracy; Figure 1 and Figure 2 only show the obvious changes in satellite orbit accuracy. Therefore, to improve the accuracy of the ultra-rapid orbit, it is necessary to analyze and correct the orbit errors of the last part of the observed orbit. For this purpose, the orbit accuracy is analyzed in the following section, in regard to two aspects, the DOP values and the observation quality.

Because the stations of the ultra-rapid-orbit determination in ACs are not publicly accessible, this study was conducted mainly based on observations of a single month (1–30 June 2016) using the data from 409 tracking stations downloaded from the AC. First, the data quality of the tracking stations observations was analyzed using a multi-GNSS data preprocessing software (MTEQC), developed and improved by the authors [28]. The MTEQC mainly refers to the data analysis and preprocessing function of the TEQC software [32], such as the cycle-slip detection. However, to analyze multi-GNSS observations (GPS/GLONASS/Galileo/BeiDou), the authors added multi-GNSS data preprocessing capability in MTEQC. Table 3 lists for all stations the average values of the 1-month data quality during the first 21 h and the last three hours over a single day in which the effective MP1 (the Multipath on P1), MP2(the Multipath on P2), and cycle-slip ratio (CSR) are listed. No significant difference is seen in the observation quality during the day.

Given that the reduced accuracy of the observed ultra-rapid orbit may arise from timeliness in the ultra-rapid-orbit determination, the observations cannot be acquired in time. Therefore, the last 3 h of observations with 200, 150, 100, and none of the 409 stations are kept in different schemes to calculate the DOP values and the corresponding orbit accuracy based on the 1-month data sets. Because the amount of experimental data and results is large, Figure 3 only plots the results of the G09, C13, E19 and R11 satellites of DOY 168, 2016, in which the DOP values and the orbit accuracy are plotted at 30-s intervals for every epoch.

From the statistics of the orbit accuracy, the orbit determined by the 409 stations was set as the reference in different schemes. Because the results are similar to the 409 stations, the scheme with 200 stations was ignored in Figure 3. Moreover, to develop the relationship between the station numbers, DOP values, and orbit accuracy, the correlation factors between the DOP values and the orbit accuracy are given in Table 4 based on the method proposed in [7,11]. However, note that there is an exponential dependence between the number of stations and the DOP values in the orbit determination [10], which is not discussed in this study. To explain this dependence, an experiment was performed to calculate the rate of change of DOP along with the number of stations based on GPS orbit determination. Figure 4 gives the relationship between the DOP values and the number stations in which the exponential dependence was found.

Based on the above experiments, the decrease in the number of stations is seen to be consistent with increasing DOP values. The orbit accuracy of the whole arc is also affected by the last 3 h of observations that arises from changes in the initial orbit parameters. Nevertheless, the DOP values are consistent with the orbit accuracy, especially for the last 3 h, the correlation factors being greater than 0.8. Therefore, the decreased accuracy of the observed ultra-rapid orbit in the last arcs arising from insufficient observations may be corrected based indirectly on the geometrical configuration of the orbit determination. The next part discusses and analyzes the orbit correction method based on the DOP values.

### 3.2. Experiments of Orbit Correction

Because the tracking-station distributions in an ultra-rapid-orbit determination is unavailable, the simulation experiments are used to verify the orbit correction method. The hourly observations steadily downloaded were merged into daily data files to incorporate them into the orbit determination. The main steps of the simulation experiments are as follows:

Step 1: Prepare the navigation files, list of stations, and station coordinates and merge daily observations (without the last 3 h of observations); in addition, all observations are preprocessed to refine the initial list of stations in the orbit determination;
Step 2: Calculate epoch-wise the DOP values of each parameter, then accumulate and add them to the orbit correction equations;
Step 3: Compare the determined and predicted ultra-rapid orbit with the multi-GNSS rapid precise orbit of GFZ to obtain the orbit residuals;
Step 4: Establish the function models between the orbit state parameters and its corresponding accumulated DOP values;
Step 5: Predict the DOP values of the last 3 h of the observed parts;
Step 6: Incorporate the predicted DOP values into the orbit correction function to correct the observed part and obtain the predicted parts.

The specific steps of the above simulation experiments are shown in Figure 5. In this study, the results of orbit corrections with 10-days (DOY 141–150, 2016) of the orbit determination were calculated. Figure 6 plots the 10-day results of the observed ultra-rapid-orbit correction during the last 3 h and the corresponding 3D RMSs before and after improvement. Furthermore, the improvement rate of the orbit accuracy before and after correction for different systems are listed in Table 5. To show more details regarding the effectiveness of the orbit correction method, the predicted DOP values (24 h) and the corrected results of C13, E19, G09, and R11 on DOY 141 were plotted (Figure 7; only the last 3 h of the observed parts are given).

From the 10-day results of the orbit correction experiments, the correction method proposed in this study based on the predicted DOP values of the last 3 h of the observed ultra-rapid orbit was found to improve the orbit accuracy by 12–22%. In addition, to present the accuracy of the observed ultra-rapid correction model, the errors for the predicted DOP values and the orbit correction model were verified. In Table 6, corresponding to Figure 7a, the differences in the 10-day DOP values between the prediction and those without the prediction (acquired based on 409 stations) for the last 3 h are given; they are presented as ratios between the average values of the prediction and calculation for the last 3 h of the observed parts.

### 3.3. Ultra-Rapid Orbit Determination

To take into account timeliness and accuracy of the ultra-rapid-orbit determination, the geometrical configuration between tracking stations and the satellites must be an optimal or sub-optimal distribution. According to previous research [10,11], there is an exponential relationship between the number of stations and the DOP values in the orbit determination. To weight the contribution of a single station in the parameter estimations, this study defines the amplification factor of the DOP values, specifically, the impact of a single station on the overall DOP values [11],

(15) $$k=[(DOP_{i}-DOP_{0})/DOP_{0}]\times 100\%\;$$

where *DOP_i_* indicates the *DOP* values based on all stations except the *i*-th station, and *DOP*_0_ represents the total DOP values before elimination. Note that, given the same distribution of stations, the more stations there are, the smaller are the DOP values. Moreover, in Equation (15), *DOP*_0_ is one more station than *DOP_i_*. Therefore, *DOP_i_* is always than *DOP*_0_ in Equation (15). Based on different amplification factors of the DOP values, the main steps to optimize the tracking stations distribution are the following:

Step 1: Obtain the initial stations list, observation files, navigation files, and the corresponding stations coordinates;
Step 2: Calculate the *DOP*_0_ values of initial stations list after data preprocessing;
Step 3: Loop all stations to output the *k_i_* (amplification factors of *i*-th station) of every station;
Step 4: Compare *k_i_* with the given *k*; if *k_i_* is greater than *k*, the corresponding station is stored in the list of stations;
Step 5: Assess whether timeliness can be meet with the requirements based on the selected list of stations; if not, continue to expand *k*;
Step 6: Output the final list of stations for orbit determination.

With this procedure, the station distribution can be optimized for ultra-rapid-orbit determination, which indirectly takes orbit accuracy and timeliness into consideration. To describe the steps in the station optimization in more detail, Figure 8 shows the experimental processes.

To analyze the effect of the station optimization methods, this study set the amplification factors to 5%, 10%, 15%, and 20%, in sequence. The list of GFZ is chosen as a reference to compare the station optimization schemes. Similarly, the observations of 409 stations downloaded by the ACs were included in the experiments. Figure 9 shows the global distributions of the different schemes. For each scheme, the orbit correction method based on the DOP values proposed in this study was used to assess the optimization strategy.

Based on the above five schemes for ultra-rapid-orbit determination, the experiments were conducted as described in Section 3.2. First, the station distributions were selected based on the different DOP-derived amplification factors. The orbit determination and correction experiments are based on a combination of 21-h observations. The accuracy of the orbit determination takes the 409 stations as references during the 24-h period to calculate the orbit accuracy in the last 3 h. In the experiments, a single month (DOY 122–151, 2016) of orbit accuracy data was obtained; the corresponding orbit 1D RMSs are plotted in Figure 10. 

The key legend for all four graphs is given in panel Figure 10d. In addition, to compare the distribution of widely used stations with that for the optimized stations, 90 stations were randomly selected and added to the orbit determination schemes.

Table 7 shows the correction results of the different satellite systems under different schemes and the corresponding number of stations. However, note that the list of stations did not change during the month for the orbit determination experiments. Moreover, the list of stations from different schemes were re-selected to ensure the reliability of the experiment results before determining orbit solutions. Moreover, in Table 7, the orbit determination of the amplification factors 5% and 10% with the same stations for BDS show an increasing 1D RMSs, which produces changes in the parameter values related to the orbit, such as the troposphere and station clocks, based on different stations of other systems.

## 4. Discussion

From the experimental results of Section 3.2, note that the orbit errors arising from the unavailability of the observations are impossible to correct completely (12–22%). On the basis of orbit determination solutions, we find two main reasons that impose limits on the observed ultra-rapid orbit correction method: (1) the correlation between the DOP values and the orbit accuracy cannot be accurately obtained and is for example affected by the perturbation models, the ambiguity resolution, and orbit determination strategies, and (2) the errors of the predicted DOP values increase gradually with increasing arc length in the last part of the observed orbit, and further limits corrections to the GNSS ultra-rapid orbit. However, the orbit determination and DOP prediction models are not the focus in orbit correction method, and will be addressed in a further study.

Moreover, it should be noted that all available stations have been included in the simulation experiments; this ensures an optimal configuration for the orbit determination given the current stations listed. However, because timeliness is restricted in the ultra-rapid-orbit determination, the geometric configuration between tracking stations and satellites does not reach optimal or sub-optimal conditions as the number of stations employed in the orbit determination diminishes, leading to a reduced correlation between the DOP values and the orbit correction. Therefore, in the orbit determination of the ultra-rapid orbit, the optimal or sub-optimal distribution of stations should be considered first.

Based on the six schemes in Section 3.3, we conclude that: (1) the effectiveness of the ultra-rapid-orbit correction decreases with increasing amplification factors of the DOP values. The orbit accuracy decreases between 1.27–6.34 cm, and the number of stations changes from 409 to 91 corresponding to the four amplification factors. This is mainly due to a decrease in the correlation between the DOP values and orbit accuracy as the number of stations decrease in the orbit determination; (2) As amplification factors increase, the number of stations gradually diminishes, with a single system being eliminated first, followed by the dual system. This is because the amplification factor defined in this study weights the contributions of each station in the multi-GNSS orbit determination; (3) By comparing the accuracy of the orbit determination with the GFZ list of stations, the correction of 111 stations was found to be better than that of 91 stations (*k* = 20%), but is worse for 101 stations (*k* = 15%); (4) In a comparison with a 90-station scheme obtained by random selection, the number of stations is comparable with that for this scheme, whereas the orbit accuracy obtained by the method in this study is higher.

From the comparisons and analyses of the six schemes, eliminating redundant information of the tracking stations based on the DOP-derived amplification factors is of significance as it ensures accuracy and timeliness of the observed ultra-rapid orbit. Based on the optimization of the station distribution, an optimal or sub-optimal relationship between the orbit accuracy and DOP values may be found that ensures a correction accuracy of the observed ultra-rapid orbit with insufficient observations.

## 5. Conclusions and Prospects

The ultra-rapid orbit is an important product for GNSS users, the accuracy of which directly affects real-time or near real-time applications. However, the orbit accuracy of the observed ultra-rapid orbit is found to diverge in the last 3-h period. Moreover, this accuracy imposes an inconvenience in the predicted ultra-rapid orbit. Based on the orbit determination, the orbit accuracy was analyzed from the perspective of data quality and availability. We concluded that the lack of observations in the last period of the observed part lowers the accuracy of the observed ultra-rapid orbit. To correct the divergence of the observed ultra-rapid orbit, this study analyzed the correlation between the DOP values and the lack of observations based on the space configuration of the orbit determination. The results show that the change in DOP values is the same as the orbit accuracy, especially for the latter period of the observed ultra-rapid orbit. Therefore, an orbit correction method based on the DOP values was proposed. In simulation experiments, the observed ultra-rapid-orbit accuracy over a 10-day period was analyzed based on the DOP values. The orbit errors arising from insufficient observations were corrected by 12–22%. However, to take into account timeliness, including all observations into orbit determination is impossible, thereby reducing the correlation between the DOP values and the orbit accuracy. Therefore, to overcome this problem, this study proposed a method to optimize the station distribution based on the timeliness and accuracy of the ultra-rapid-orbit determination. An amplification factor for the DOP values was defined using the contribution of a single station. Moreover, we used in the experiments four schemes to select stations, the GFZ list of stations, and 90 randomly selected stations; the results show that the station optimization proposed in this study is an optimal or sub-optimal method when the number of stations is the same. The orbit accuracy in this instance is better than that of the GFZ list of stations with a smaller number of stations.

The orbit accuracy is influenced by many factors, including perturbation models used, parameter estimation models, and GPS ambiguity resolution. Moreover, the spatial configuration of satellites and stations cannot reflect the orbit accuracy completely. Therefore, further research to refine the correction models is needed in regard to other influencing factors in orbit determination.

## Figures and Tables

**Figure 1:**
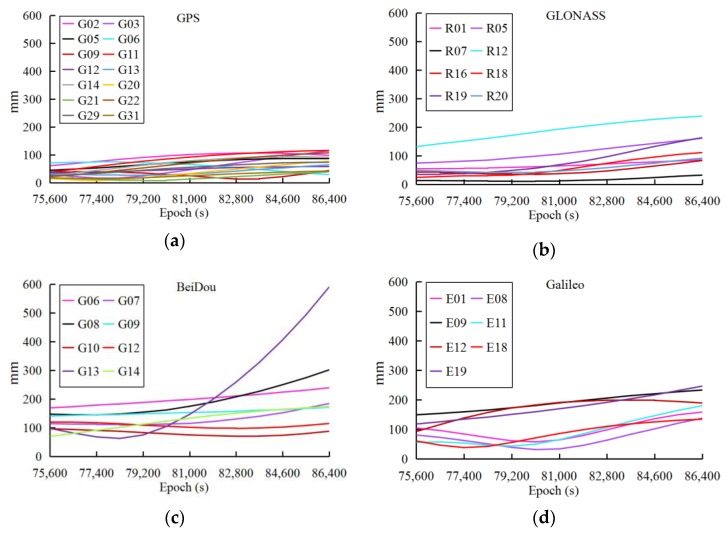
3D RMSs between the observed ultra-rapid orbit of WHU and the rapid orbit of GFZ during the last three hours: (**a**) GPS, (**b**) GLONASS, (**c**) BeiDou, (**d**) Galileo.

**Figure 2:**
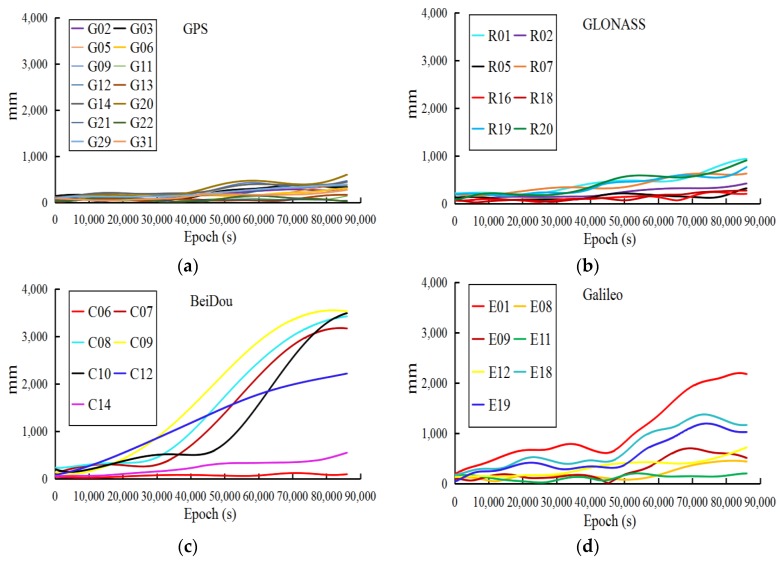
3D RMSs between the predicted ultra-rapid orbit based on WHU and the rapid orbit of GFZ during 24 h: (**a**) GPS, (**b**) GLONASS, (**c**) BeiDou, (**d**) Galileo.

**Figure 3:**
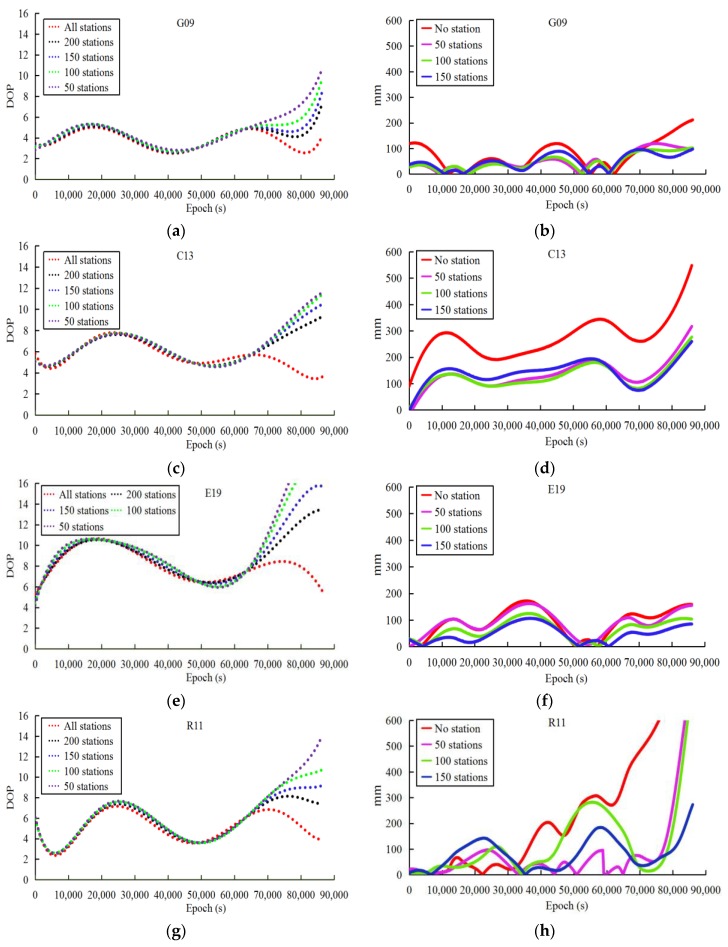
DOP values and orbit accuracy of different schemes with different number of stations of last 3 h for each epoch (30 s interval: (**a**,**b**) G09, (**c**,**d**) C13, (**e**,**f**) E19, (**g**,**h**) R11).

**Figure 4:**
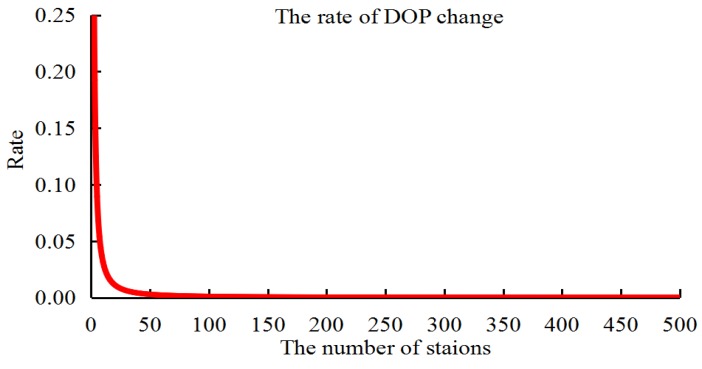
Relationship between the rate of DOP change and the number of stations.

**Figure 5:**
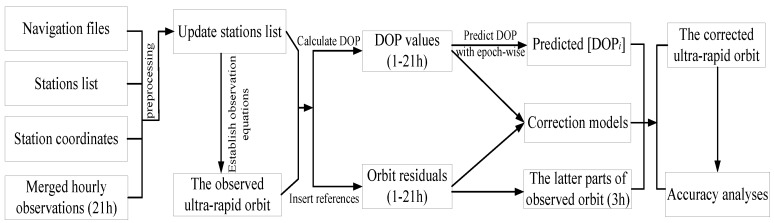
Flowchart of orbit correction method based on DOP values.

**Figure 6:**
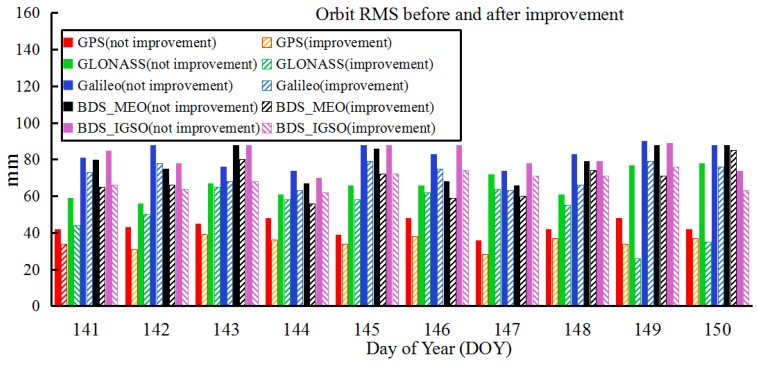
10-day (DOY 141–150, 2016) 3D RMSs of the GNSS observed orbit for the last 3 h based on improvement and not improvement method.

**Figure 7:**
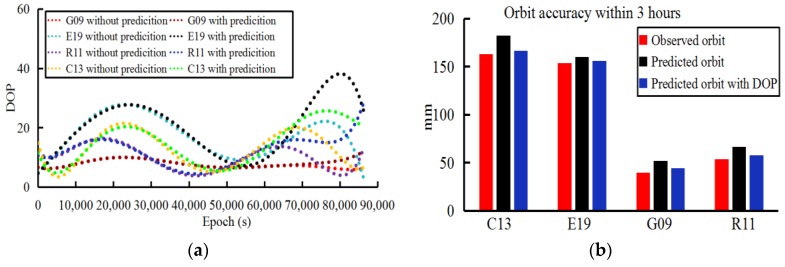
Orbit accuracy before and after correction of the last three hours and the corresponding DOP values on DOY 141: (**a**) DOP values with and without prediction, (**b**) orbit accuracy based on observed, predicted and correction, respectively.

**Figure 8:**
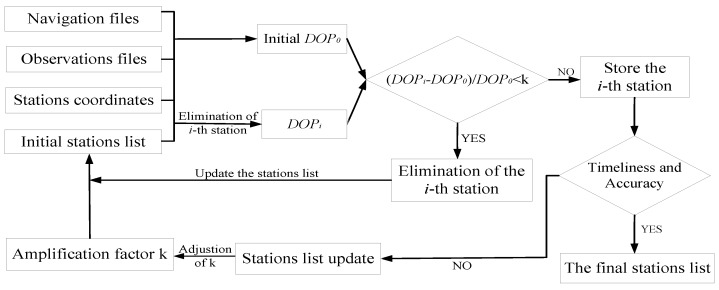
Flowchart of the station-distribution optimization based on DOP values.

**Figure 9:**
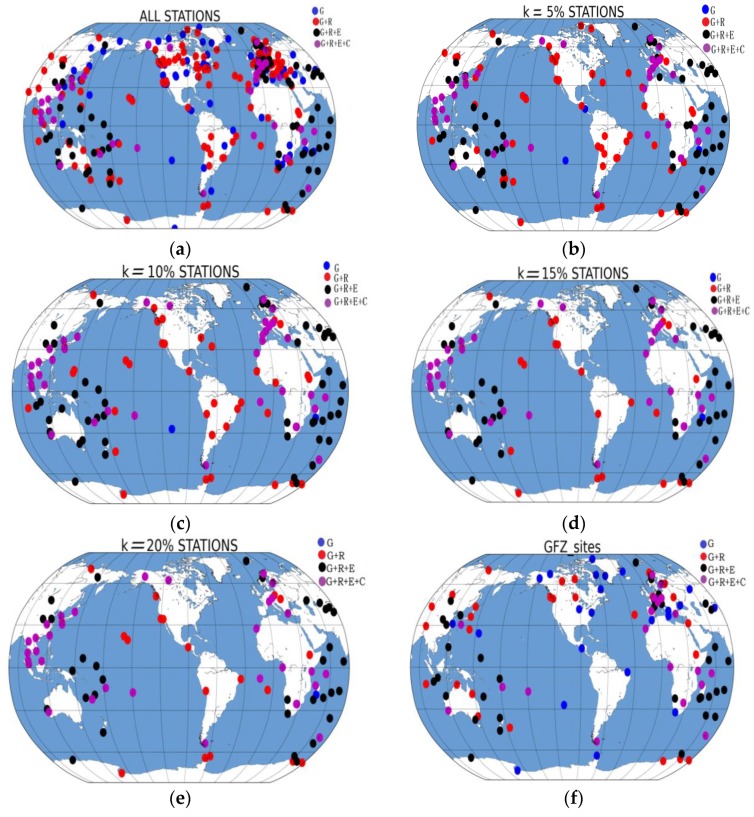
Distributions of stations for the different schemes: (**a**) all stations, (**b**) 5%, (**c**) 10%, (**d**) 15%, (**e**) 20%, (**f**) GFZ_sites.

**Figure 10:**
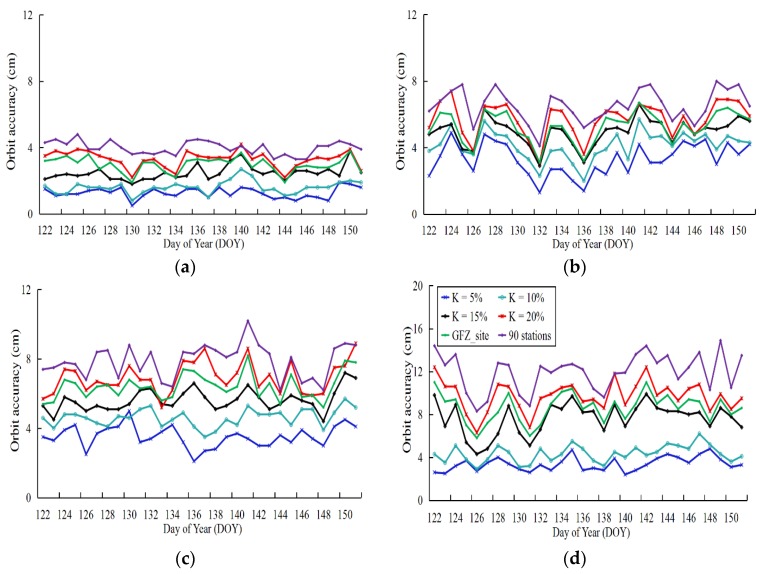
Orbit 1D RMSs of different orbit determination schemes: (**a**) GPS, (**b**) GLONASS, (**c**) Galileo, (**d**) BeiDou.

**Table 1:** Average 3D RMSs of the observed ultra-rapid orbit (cm) of WHU AC.

Systems	1–20 h	21 h	22 h	23 h	24 h
GPS	3.6	3.5	4.6	6.3	7.5
GLONASS	6.1	5.6	6.1	7.5	10.2
BeiDou	12.9	12.1	12.4	15.6	21.8
Galileo	8.2	13.2	13.5	14.4	17.9

**Table 2:** Average 3D RMSs of the predicted ultra-rapid orbit (cm) based on WHU AC.

Systems	2 h	4 h	6 h	1–12 h	1–24 h
GPS	7.8	9.8	10.6	10.8	16.1
GLONASS	12.5	13.4	13.6	14.3	21.9
BeiDou	23.2	39.9	61.1	70.1	139.9
Galileo	16.9	26.6	32.5	34.4	49.9

**Table 3:** Data quality of one-month observations over a single day.

		Effective (%)	MP1 (m)	MP2 (m)	CSR
1–21 h	Minimum	96.8	0.04	0.03	0.02
	Maximum	100.0	0.28	0.31	4.22
	Average	95.1	0.22	0.26	1.49
21–24 h	Minimum	94.2	0.06	0.09	0.02
	Maximum	100.0	0.28	0.41	4.81
	Average	96.3	0.21	0.38	2.48

**Table 4:** Correlation factors between the DOP values and orbit accuracy for different schemes.

Station Numbers	Correlation Factors
409	0.9283
200	0.8846
150	0.8635
100	0.8134
0	-

**Table 5:** 10-day (DOY 141–150, 2016) results (mm) of orbit accuracy for the last three hours based on improvement and no improvement method and its improvement rate.

Systems	141	142	143	144	145	146	147	148	149	150	Improvement Rate
GPS (no improvement)	42	43	45	48	39	48	36	42	48	42	-
GPS (improvement)	34	31	39	36	34	38	28	37	34	37	20%
GLONASS (no improvement)	59	56	67	61	66	66	72	61	77	78	-
GLONASS (improvement)	44	50	65	58	58	62	64	55	26	35	22%
Galileo (no improvement)	81	88	76	74	88	83	74	83	90	88	-
Galileo (improvement)	73	78	68	63	79	75	63	66	79	76	13%
BDS_MEO (no improvement)	80	75	88	67	86	68	66	79	88	88	-
BDS_MEO (improvement)	65	66	80	56	72	59	60	74	71	85	12%
BDS_IGSO (no improvement)	85	78	88	70	88	88	78	79	89	74	-
BDS_IGSO (improvement)	66	64	68	62	72	74	71	71	76	63	16%

**Table 6:** Ratio between the prediction and without prediction of the last three hours.

Satellites	141	142	143	144	145	146	147	148	149	150
G09	94%	90%	92%	88%	89%	92%	93%	94%	88%	90%
R11	79%	76%	76%	82%	86%	82%	76%	76%	68%	86%
C13	78%	68%	68%	84%	72%	63%	67%	78%	79%	70%
E19	76%	66%	62%	62%	64%	56%	66%	67%	60%	76%

**Table 7:** Orbits 1D RMS (cm) of the last 3 h before and after correction and the corresponding station numbers for different schemes.

Schemes	GPS		GLONASS		Galileo		BeiDou	
	ORB (cm) 1D RMSs	Number of Stations	ORB (cm) 1D RMSs	Number of Stations	ORB (cm) 1D RMSs	Number of Stations	ORB (cm) 1D RMSs	Number of Stations
All stations	-	409	-	281	-	118	-	38
5%	1.27	171	3.31	165	3.53	101	3.38	38
10%	1.61	131	4.14	129	4.61	92	4.33	38
15%	2.48	101	4.95	100	5.63	76	7.63	35
20%	3.30	91	5.72	90	6.93	70	9.72	34
GFZ_site	2.97	111	5.25	106	6.42	76	8.62	20
90 stations	3.99	90	6.53	90	7.89	66	11.95	26
